# Toeplitz–Hankel Structured Covariance Reconstruction for DOA Estimation of Coherent Sources with Coprime Arrays Under Nonuniform Noise

**DOI:** 10.3390/s26165041

**Published:** 2026-08-08

**Authors:** Heng Zhao, Ying Hu, Zijing Zhang, Fei Zhang

**Affiliations:** 1 Ocean College, Jiangsu University of Science and Technology, Zhenjiang 212100, China; 232241803238@stu.just.edu.cn (H.Z.); zjzf@just.edu.cn (F.Z.); 2 School of Instrument Science and Engineering, Southeast University, Nanjing 210096, China; 230228943@seu.edu.cn

**Keywords:** direction-of-arrival estimation, coprime array, coherent sources, nonuniform noise, Toeplitz covariance reconstruction, Hankel low-rank refinement, MUSIC, virtual array

## Abstract

Direction-of-arrival (DOA) estimation with coprime arrays can synthesize an enlarged virtual aperture from a limited number of physical sensors. However, coherent incident sources lead to rank deficiency of the source covariance matrix, while unknown nonuniform sensor noise mainly contaminates the zero-lag component of the difference-coarray covariance. These two effects jointly degrade conventional Coarray Root-MUSIC, Coarray ESPRIT, and interpolation-based virtual-array methods. To address this problem, this paper proposes a Toeplitz–Hankel structured covariance reconstruction method for coherent-source DOA estimation with coprime arrays under unknown nonuniform noise. The method first performs redundancy-aware difference-coarray lag averaging. The zero-lag component is then suppressed during missing-lag interpolation to reduce the bias caused by sensor-dependent noise powers. A Toeplitz positive semidefinite projection is used to enforce covariance validity, and a relaxed Hankel truncated-singular-value-decomposition refinement is introduced to enhance the low-rank spectral structure of the reconstructed virtual covariance sequence. Finally, multi-scale forward–backward spatial smoothing MUSIC is applied for coherent-source DOA estimation. Simulation results with a coprime array of M=4 and N=5 show that the proposed method provides more accurate and stable DOA estimates than Coarray Root-MUSIC, Coarray ESPRIT, and RV-TSI. Compared with CVX-based THSCR, the proposed method avoids semidefinite programming and nuclear-norm optimization and reduces the average runtime from approximately 11.2 s per trial to approximately 0.11 s per trial under the tested setting.

## 1. Introduction

Direction-of-arrival (DOA) estimation is a fundamental task in array signal processing and is widely used in radar, sonar, wireless communications, acoustic localization, and intelligent sensing systems. Classical subspace-based algorithms, such as MUSIC [1] and ESPRIT [2], exploit the structure of the array covariance matrix to obtain high-resolution angular estimates. Root-MUSIC further transforms the spectral search of MUSIC into a polynomial-rooting problem and is often used as a strong subspace-based baseline for uniform virtual arrays [3]. A comprehensive discussion of array signal processing and spectral estimation theory can be found in [4,5,6]. These methods usually assume that the array covariance matrix is accurately estimated, that the source covariance matrix has full rank, and that the additive noise is spatially white or has been properly calibrated. In practical sensing environments, however, these assumptions are often violated by coherent multipath propagation, limited snapshots, and sensor-dependent nonuniform noise.

Sparse arrays provide an effective way to enlarge the available aperture without increasing the number of physical sensors. Nested arrays [7] and coprime arrays [8,9] can generate a difference coarray with many more virtual lags than physical sensors. By averaging the physical covariance entries associated with identical lags, a sparse physical array can synthesize a virtual uniform linear array (ULA). This property increases the degrees of freedom and improves angular resolution. The super-resolution viewpoint of coprime-array DOA estimation has also been studied in [10]. Nevertheless, the difference-coarray covariance is usually incomplete, and missing virtual lags must be reconstructed before standard ULA-based subspace estimation can be applied.

The coexistence of coherent sources and unknown nonuniform noise makes the reconstruction problem more difficult. Coherent sources cause the source covariance matrix to become rank deficient or nearly rank deficient. As a result, direct MUSIC processing may produce merged or broadened spectral peaks. Spatial smoothing can decorrelate coherent sources, but its performance depends strongly on whether the reconstructed virtual covariance matrix preserves a valid Toeplitz covariance structure [11,12]. Hu et al. exploited fourth-order cumulants for coherent-signal DOA estimation with coprime arrays [13]. More recently, Ma et al. developed coherent-signal DOA estimation methods from the perspective of signal-subspace reconstruction, where Hankel matrix construction and rank recovery are used to restore the source-related subspace structure [14]. These studies demonstrate the importance of structural processing for coherent-source estimation. However, they do not explicitly target the joint problem of coherent sources and unknown nonuniform sensor noise.

Unknown nonuniform noise introduces another important difficulty. Since the diagonal entries of the physical covariance matrix are mapped to the zero-lag component in the coarray domain, the zero-lag covariance contains not only source power but also unknown sensor-dependent noise power. If this contaminated zero-lag component is directly used for interpolation or structured reconstruction, the reconstructed virtual covariance matrix may be biased. Therefore, it is necessary to distinguish the zero-lag component from the nonzero-lag components when reconstructing the virtual covariance under unknown nonuniform noise. In practical radar systems, calibration errors and polarimetric echo distortions may also lead to channel-dependent perturbations. Xu et al. discussed full-polarization radar target measurement and calibration at subterahertz frequencies, highlighting the importance of polarimetric echo errors and calibration in practical radar measurements [15]. These effects further justify modeling sensor-dependent nonuniform noise in the covariance domain.

Structured covariance reconstruction has therefore become an important tool for sparse-array DOA estimation. Toeplitz reconstruction enforces the covariance structure of a virtual ULA, while Hankel low-rank enhancement exploits the spectral sparsity of the covariance sequence. Positive definite Toeplitz completion and sparse covariance-based estimation provide important theoretical foundations for covariance reconstruction in nonuniform arrays and source localization problems [16,17,18,19]. Rank-minimization-based Toeplitz reconstruction has been investigated for coprime-array DOA estimation [20]. Coarray ESPRIT-type processing has also been used to exploit the shift-invariance property of the virtual ULA [21]. In addition, alternating-projection-based gridless covariance estimation provides another way to impose covariance-domain constraints without conventional grid search [22]. Convex optimization methods based on semidefinite programming (SDP), trace minimization, reweighting, and nuclear-norm recovery can obtain high-quality covariance matrices, but they require repeated optimization and are computationally expensive. This high computational burden is especially unfavorable for Monte Carlo evaluation, embedded implementation, or real-time localization.

Recent radar-oriented DOA studies have also extended direction finding to more complex propagation and sensing scenarios. For example, Xie et al. investigated NLOS direction finding in RIS-aided bistatic MIMO radar through covariance tensor decomposition [23]. This work shows that structured covariance-domain processing is also useful in advanced radar systems with non-line-of-sight propagation and reconfigurable intelligent surfaces. Such studies further highlight the importance of robust covariance modeling and reconstruction for practical DOA estimation.

In addition to covariance-reconstruction-based DOA estimation, recent AoA-based localization studies further show the practical importance of efficient angular estimation. Florio et al. investigated the localization of UHF RFID tags through real-time AoA estimation and developed a dedicated digital architecture for phase-interferometry-based RFID localization [24]. Böller et al. combined time-of-flight and AoA measurements for SHF RFID-based localization, demonstrating that AoA information can support realistic tracking of RFID transponders and tagged objects [25]. These studies indicate that DOA/AoA estimation is not only a theoretical array-processing problem but also a key component in practical localization, tracking, and intelligent sensing applications.

At the same time, practical AoA systems are often constrained by hardware cost, sampling resources, and real-time processing requirements. Florio et al. proposed a low-complexity AoA estimation system using 1-bit conversion, where the objective is to avoid a full high-resolution ADC stage and support real-time AoA measurement through a hardware-friendly architecture [26]. Such works highlight the need for DOA/AoA algorithms that reduce computational complexity while preserving sufficient estimation accuracy.

Recently, Pan et al. proposed a real-valued DOA estimation method for coprime arrays via Toeplitz-construction-based spline interpolation (RV-TSI) [27]. This method converts the virtual covariance reconstruction problem into a real-valued spline interpolation task and therefore significantly reduces the computational complexity compared with matrix-completion or convex-optimization methods. However, interpolation alone does not explicitly enforce positive semidefinite covariance validity and Hankel low-rank spectral structure, which may limit its robustness under coherent sources and unknown nonuniform noise. Our previous work developed a CVX-based Toeplitz–Hankel structured covariance reconstruction (THSCR) framework to jointly handle coherent sources and unknown nonuniform noise through Toeplitz positive semidefinite recovery and Hankel low-rank restoration [28]. Although good estimation performance was achieved, the method depends on SDP-based Toeplitz reconstruction and Hankel nuclear-norm optimization, leading to relatively high computational complexity.

Motivated by these observations, an optimization-free reconstruction strategy is introduced to reduce the computational burden of the previous CVX-based THSCR framework. Specifically, SDP-based Toeplitz recovery and Hankel nuclear-norm optimization are replaced by PCHIP missing-lag interpolation, EVD-based Toeplitz PSD projection, and truncated-SVD Hankel refinement. This design avoids repeated convex optimization and is more suitable for computationally constrained applications, such as automotive millimeter-wave radar perception, UAV or mobile-robot sensing, and coastal radar monitoring, where DOA estimates need to be updated repeatedly or in near real time.

The main contributions are summarized as follows:

1. A computation-efficient Toeplitz–Hankel structured covariance reconstruction framework is developed for coherent-source DOA estimation with coprime arrays under unknown nonuniform noise. The proposed framework preserves the key covariance-domain constraints of Toeplitz validity, Hankel spectral structure, zero-lag suppression, and FBSS-based decorrelation. Therefore, it reduces the implementation burden while maintaining strong robustness against coherent-source rank deficiency, missing coarray lags, finite-snapshot covariance errors, and sensor-dependent nonuniform noise.
2. An optimization-free reconstruction strategy is introduced to reduce the computational burden of the previous CVX-based THSCR framework without discarding the essential structural constraints. Specifically, SDP-based Toeplitz recovery and Hankel nuclear-norm optimization are replaced by PCHIP missing-lag interpolation, EVD-based Toeplitz PSD projection, and truncated-SVD Hankel refinement. This design avoids repeated convex optimization, greatly improves computational efficiency, and remains robust because the reconstructed covariance matrix is still constrained by Toeplitz PSD validity and Hankel low-rank spectral enhancement.
3. A multi-scale FBSS-MUSIC estimator is integrated with the accelerated reconstruction pipeline to improve coherent-source decorrelation and spectral stability. Extensive simulations are conducted to evaluate estimation accuracy, nonuniform-noise robustness, source-number influence, MUSIC spectra, scatter distribution, runtime, and computational complexity. The results demonstrate that the proposed method achieves a favorable balance between low computational cost, DOA estimation accuracy, and robustness compared with Coarray Root-MUSIC, Coarray ESPRIT, RV-TSI, and CVX-based THSCR.

The remainder of this paper is organized as follows. Section 2 presents the signal model and the coprime-array difference-coarray model. Section 3 describes the proposed Toeplitz–Hankel structured covariance reconstruction method in detail. Section 4 gives the simulation setup. Section 5 analyzes the numerical results. Section 6 derives the computational complexity formulas. Section 7 concludes the paper.

## 2. Signal Model and Difference-Coarray Representation

### 2.1. Coprime-Array Geometry

Consider a coprime array constructed from two sparse ULAs. Let *M* and *N* be two coprime positive integers. The physical sensor-position set normalized by half-wavelength spacing is defined as

(1) P={0,M,2M,…,(N−1)M}∪{0,N,2N,…,(M−1)N}.

After removing the repeated sensor at the origin, the number of distinct physical sensors is L=M+N−1. In this paper, the simulation array uses M=4 and N=5, yielding

(2) P={0,4,5,8,10,12,15,16},L=8.

Although only eight physical sensors are used, the pairwise difference set generates a larger number of virtual lags, thereby increasing the effective aperture used for DOA estimation.

### 2.2. Received Signal Model

Assume that *K* narrowband far-field sources impinge on the array from the directions

(3) $$\theta ={\left[\theta_{1},\theta_{2},\ldots ,\theta_{K}\right]}^{T}.$$

The received signal vector at snapshot *t* is modeled as

(4) x(t)=A(θ)s(t)+n(t),t=1,2,…,T,

where $x\left(t\right)\in C^{L\times 1}$ is the received data vector, $s\left(t\right)\in C^{K\times 1}$ is the source waveform vector, $n\left(t\right)\in C^{L\times 1}$ is the additive noise vector, and *T* is the number of snapshots. The steering matrix is

(5) $$A\left(\theta \right)=[a\left(\theta_{1}\right),a\left(\theta_{2}\right),\ldots ,a\left(\theta_{K}\right)],$$

with the steering vector

(6) $$a\left(\theta_{k}\right)={\left[e^{-j\pi p_{1}\sin\theta_{k}},e^{-j\pi p_{2}\sin\theta_{k}},\ldots ,e^{-j\pi p_{L}\sin\theta_{k}}\right]}^{T},$$

where $p_{l}\in P$ denotes the *l*th physical sensor position.

The theoretical covariance matrix of the physical array is

(7) $$R_{x}=E\left\{x\left(t\right)x^{H}\left(t\right)\right\}=AR_{s}A^{H}+R_{n},$$

where $R_{s}=E\left\{s\left(t\right)s^{H}\left(t\right)\right\}$ is the source covariance matrix and $R_{n}=E\left\{n\left(t\right)n^{H}\left(t\right)\right\}$ is the noise covariance matrix. The sample covariance matrix is

(8) $${\hat{R}}_{x}=\frac{1}{T}\sum_{t=1}^{T}x\left(t\right)x^{H}\left(t\right)=\frac{1}{T}XX^{H}.$$

### 2.3. Coherent-Source Model

In the simulations, coherent sources are generated by using one common waveform component and independent perturbation components. A representative model is

(9) $$s_{1}\left(t\right)=u_{0}\left(t\right),$$

(10) $$s_{k}\left(t\right)=\rho u_{0}\left(t\right)+\sqrt{1-\rho^{2}}\;u_{k}\left(t\right),\;k=2,3,\ldots ,K,$$

where $u_{0}\left(t\right)$ and $u_{k}\left(t\right)$ are independent complex Gaussian sequences with unit power and ρ∈[0,1] is the coherence coefficient. When ρ approaches one, different source waveforms become highly correlated and $R_{s}$ becomes ill-conditioned or rank deficient. This rank deficiency destroys the direct signal/noise subspace separation and motivates the use of FBSS after virtual covariance reconstruction.

### 2.4. Unknown Nonuniform Noise Model

The additive noise is assumed to be spatially independent but nonuniform across sensors:

(11) $$R_{n}=\mathrm{diag}\left\{\sigma_{1}^{2},\sigma_{2}^{2},\ldots ,\sigma_{L}^{2}\right\}.$$

In the simulation code, the average noise power is determined by the SNR, and the nonuniform profile is generated as

(12) $$\eta_{l}=1+\alpha {\left(\frac{l-1}{L-1}\right)}^{2},\;l=1,2,\ldots ,L,$$

(13) $${\bar{\eta }}_{l}=\frac{\eta_{l}}{\frac{1}{L}\sum_{q=1}^{L}\eta_{q}},\;\sigma_{l}^{2}=\sigma_{n}^{2}{\bar{\eta }}_{l},$$

where α controls the degree of nonuniformity. Thus, the noise covariance is diagonal but not proportional to the identity matrix. In the difference-coarray domain, diagonal entries of ${\hat{R}}_{x}$ correspond to zero lag, so the zero-lag value contains the sum of source power and nonuniform sensor noise power. In contrast, nonzero lags are not directly contaminated by the diagonal noise powers. This observation is the basis of the zero-lag suppression step in the proposed method.

### 2.5. Difference-Coarray Covariance Model

For any sensor pair (i,j), the covariance entry can be written as

(14) $${\left[R_{x}\right]}_{ij}=\sum_{k=1}^{K}\sum_{q=1}^{K}{\left[R_{s}\right]}_{kq}e^{-j\pi (p_{i}\sin\theta_{k}-p_{j}\sin\theta_{q})}+{\left[R_{n}\right]}_{ij}.$$

For uncorrelated sources, the covariance entry depends on the position difference $p_{i}-p_{j}$. For coherent sources, cross-terms exist, but the coarray covariance sequence still contains the angular information and can be processed after structured reconstruction and smoothing.

The difference-coarray lag set is

(15) $$D=\{l=p_{i}-p_{j}|p_{i},p_{j}\in P\}.$$

For each lag *ℓ*, the redundancy count is

(16) $$c_{l}=\left|\{\left(i,j\right)|p_{i}-p_{j}=l\}\right|.$$

The observed virtual covariance at lag *ℓ* is obtained by redundancy-aware averaging:

(17) $$\hat{r}\left(l\right)=\frac{1}{c_{l}}\sum_{p_{i}-p_{j}=l}{\left[{\hat{R}}_{x}\right]}_{ij},\;l\in D.$$

To reduce finite-snapshot asymmetry, Hermitian averaging is further imposed as

(18) $${\hat{r}}_{h}\left(l\right)=\frac{1}{2}\left[\hat{r}\left(l\right)+{\hat{r}}^{*}\left(-l\right)\right],\;l>0.$$

The nonnegative-lag sequence ${\left[{\hat{r}}_{h}\left(0\right),{\hat{r}}_{h}\left(1\right),\ldots ,{\hat{r}}_{h}\left(Q-1\right)\right]}^{T}$ is then used to construct a virtual ULA covariance matrix after missing-lag reconstruction.

## 3. Proposed Method

The proposed method is designed according to three practical observations. First, redundant coarray lags should be averaged to reduce covariance estimation fluctuation. Second, the zero-lag component should not dominate interpolation because it is biased by unknown nonuniform noise. Third, the reconstructed covariance should satisfy both Toeplitz PSD covariance validity and Hankel low-rank spectral structure. Based on these observations, the proposed method processes the data in a sequential manner. It first estimates the physical sample covariance matrix and maps it to the difference-coarray domain through redundancy-aware lag averaging. Then, the zero-lag covariance is excluded from the missing-lag interpolation stage to suppress the bias caused by sensor-dependent noise powers. After the missing nonzero lags are recovered, the virtual covariance sequence is converted into a Toeplitz matrix and projected onto the PSD cone so that it satisfies the basic physical requirements of a covariance matrix. Next, a Hankel matrix is constructed from the Toeplitz covariance sequence, and truncated SVD is used to retain the dominant exponential components associated with the incident sources. Finally, the refined covariance matrix is processed by multi-scale FBSS-MUSIC to decorrelate coherent sources and obtain the DOA estimates. In this way, the proposed method keeps the key structural constraints of CVX-based Toeplitz–Hankel reconstruction, but implements them through direct interpolation, projection, SVD, and subspace-search operations rather than through SDP or nuclear-norm optimization.

### 3.1. Zero-Lag Suppressed Missing-Lag Interpolation

Let Ω be the set of observed nonzero lags after coarray averaging:

(19) Ω={l∈D∣l≠0}.

The zero lag is excluded from the interpolation set because

(20) $$\hat{r}\left(0\right)=\frac{1}{L}\sum_{i=1}^{L}{\left[{\hat{R}}_{x}\right]}_{ii}\approx \frac{1}{L}\sum_{i=1}^{L}{\left[AR_{s}A^{H}\right]}_{ii}+\frac{1}{L}\sum_{i=1}^{L}\sigma_{i}^{2},$$

which contains the unknown average noise power. Therefore, directly using $\hat{r}\left(0\right)$ in interpolation may enlarge the zero-lag magnitude and distort the reconstructed Toeplitz covariance.

For each missing lag l∉Ω, the real and imaginary parts of the virtual covariance are interpolated separately. Here, $I_{\mathrm{pchip}}$ denotes the piecewise cubic Hermite interpolating polynomial interpolation operator. It is used to recover the missing virtual covariance lags from the observed nonzero-lag samples. Compared with high-order polynomial interpolation, PCHIP is shape-preserving and can reduce artificial oscillations in the reconstructed covariance sequence, which helps preserve the stability of the subsequent Toeplitz covariance construction.

(21) $${\tilde{r}}_{R}\left(l\right)=I_{\mathrm{pchip}}\left\{\left(q,ℜ\left[\hat{r}\left(q\right)\right]\right),q\in \Omega \right\},$$

(22) $${\tilde{r}}_{I}\left(l\right)=I_{\mathrm{pchip}}\left\{\left(q,ℑ\left[\hat{r}\left(q\right)\right]\right),q\in \Omega \right\},$$

(23) $$\tilde{r}\left(l\right)={\tilde{r}}_{R}\left(l\right)+j{\tilde{r}}_{I}\left(l\right).$$

The shape-preserving interpolation avoids the oscillatory behavior that may occur in high-order polynomial interpolation. After interpolation, conjugate symmetry is restored as

(24) $$\tilde{r}\left(l\right)\leftarrow \frac{1}{2}\left[\tilde{r}\left(l\right)+{\tilde{r}}^{*}\left(-l\right)\right],\;\tilde{r}\left(-l\right)={\tilde{r}}^{*}\left(l\right).$$

The zero-lag value is then reconstructed by a bounded replacement strategy rather than direct use of the observed diagonal-contaminated value. Define

(25) $$r_{\max}=\max_{l\neq 0}\left|\tilde{r}\left(l\right)\right|.$$

The lower and upper bounds of the zero-lag value are set as

(26) $$r_{0,\min}=\gamma_{1}r_{\max},\;r_{0,\max}=\gamma_{2}r_{\max},$$

where $\gamma_{1}>1$ and $\gamma_{2}>\gamma_{1}$ are constants. In the implementation, $\gamma_{1}=1.08$ and $\gamma_{2}=1.80$. If the observed zero-lag value is too small or too large, it is compressed into the reliable range. This strategy preserves the positive energy role of the zero-lag component while preventing it from being dominated by unknown noise.

### 3.2. Toeplitz Covariance Construction and PSD Projection

After interpolation, the nonnegative-lag sequence is written as

(27) $$\tilde{c}={\left[\tilde{r}\left(0\right),\tilde{r}\left(1\right),\ldots ,\tilde{r}\left(Q-1\right)\right]}^{T}.$$

The initial Toeplitz covariance matrix is constructed by

(28) $$\tilde{T}=\mathrm{Toep}\left(\tilde{c}\right),\;{\left[\tilde{T}\right]}_{ij}=\tilde{r}\left(i-j\right).$$

A valid covariance matrix should be Hermitian PSD. However, interpolation and finite-snapshot perturbation may produce negative eigenvalues. Therefore, the proposed method alternates between PSD projection and Toeplitz projection. First, eigendecomposition is performed:

(29) $$\tilde{T}=U\Lambda U^{H}.$$

The eigenvalues are floored as

(30) $$\Lambda_{+}=\mathrm{diag}\left\{\max\left(\lambda_{1},\epsilon \right),\ldots ,\max\left(\lambda_{Q},\epsilon \right)\right\},$$

where ϵ is a small positive constant. The PSD-projected matrix is

(31) $$T_{+}=U\Lambda_{+}U^{H}.$$

Toeplitz projection is then applied by diagonal averaging:

(32) $${\left[T\left(T_{+}\right)\right]}_{ij}=\frac{1}{Q-|i-j|}\sum_{m-n=i-j}{\left[T_{+}\right]}_{mn}.$$

The PSD and Toeplitz projections are repeated for a small number of iterations. This stage replaces the CVX-based Toeplitz PSD optimization while retaining the main covariance constraint.

### 3.3. Hankel Truncated-SVD Refinement

Let $c_{p}$ denote the first column of the projected Toeplitz covariance matrix. The Hankel matrix is constructed as

(33) $$H=\mathrm{Hankel}\left(c_{p}\right),\;{\left[H\right]}_{ij}=c_{p,i+j-1},$$

where $i=1,2,\ldots ,L_{h}$, $j=1,2,\ldots ,R_{h}$, and

(34) $$L_{h}=\left\lfloor \frac{Q}{2}\right\rfloor +1,\;R_{h}=Q-L_{h}+1.$$

For a small number of sources, the covariance sequence is composed of a limited number of complex exponentials, and the corresponding Hankel matrix has an approximate low-rank structure. Instead of solving a nuclear-norm minimization problem, the proposed method computes

(35) $$H=U_{h}\Sigma_{h}V_{h}^{H},$$

retains the dominant $r_{h}=K+1$ singular components, and obtains

(36) $$H_{r_{h}}=U_{h}\left(:,1:r_{h}\right)\Sigma_{h}\left(1:r_{h},1:r_{h}\right)V_{h}^{H}\left(:,1:r_{h}\right).$$

The refined covariance sequence is recovered by anti-diagonal averaging:

(37) $${\hat{c}}_{m}=\frac{1}{|A_{m}|}\sum_{\left(i,j\right)\in A_{m}}{\left[H_{r_{h}}\right]}_{ij},\;A_{m}=\left\{\left(i,j\right)|i+j-1=m\right\}.$$

To avoid over-smoothing, the final sequence is obtained by convex mixing:

(38) $$c_{f}=\beta \hat{c}+\left(1-\beta \right)c_{p},$$

where 0<β<1. In the implementation, β=0.25. The parameter β controls the strength of the Hankel truncated-SVD refinement. A larger β gives more weight to the low-rank Hankel approximation and can suppress residual perturbations more strongly, but it may also over-smooth the covariance sequence and weaken useful angular information. A smaller β preserves more details from the Toeplitz-projected covariance, but the residual noise and interpolation errors may not be sufficiently reduced. Therefore, a moderate value of β=0.25 is used to balance low-rank enhancement and covariance-detail preservation. The final reconstructed covariance matrix is

(39) $$T_{f}=\mathrm{Toep}\left(c_{f}\right).$$

### 3.4. Multi-Scale FBSS-MUSIC

Coherent sources reduce the rank of the signal covariance matrix. Therefore, FBSS is applied to $T_{f}$. For a smoothing subarray length *p*, the forward-smoothed covariance is

(40) $$R_{f}=\frac{1}{Q-p+1}\sum_{i=1}^{Q-p+1}J_{i}T_{f}J_{i}^{T},$$

where $J_{i}$ selects the *i*th contiguous subarray of length *p*. The backward-smoothed covariance is incorporated through

(41) $$R_{\mathrm{fb}}=\frac{1}{2}\left(R_{f}+\Pi R_{f}^{*}\Pi \right),$$

where Π is the exchange matrix. To reduce dependence on a single *p*, several ratios are used:

(42) $$p_{s}=\left\lfloor \eta_{s}Q\right\rceil ,\;\eta_{s}\in \left\{0.80,0.88,0.95\right\}.$$

For each $p_{s}$, eigendecomposition of $R_{\mathrm{fb}}^{\left(s\right)}$ yields the noise subspace $E_{n}^{\left(s\right)}$. The MUSIC spectrum is

(43) $$P_{s}\left(\theta \right)=\frac{1}{a_{p}^{H}\left(\theta \right)E_{n}^{\left(s\right)}E_{n}^{(s)H}a_{p}\left(\theta \right)},$$

where $a_{p}\left(\theta \right)={\left[1,e^{-j\pi \sin\theta },\ldots ,e^{-j\pi (p_{s}-1)\sin\theta }\right]}^{T}$. The final accumulated spectrum is

(44) $$P\left(\theta \right)=\sum_{s=1}^{S}\frac{P_{s}\left(\theta \right)}{\max_{\theta }P_{s}\left(\theta \right)},$$

where S=3 in the implementation.The use of three FBSS scales is a trade-off between robustness and computational cost. A single smoothing length may make the final MUSIC spectrum sensitive to the selected subarray size. By contrast, using multiple smoothing lengths can stabilize the spectrum under coherent sources. However, using too many scales increases the runtime because each scale requires smoothing, eigendecomposition, and spectral search. Therefore, three representative scales are used to improve robustness while keeping the method suitable for low-complexity implementation. The estimated DOAs are obtained by peak searching:

(45) $$\hat{\theta }={\mathrm{PeakSearch}}_{K}\left\{P\left(\theta \right),\theta \in \Theta \right\},$$

followed by local grid refinement around each coarse peak.

The above processing stages jointly constitute the proposed Toeplitz–Hankel structured covariance reconstruction framework. For clarity, the complete implementation procedure is summarized in Algorithm 1.
**Algorithm 1** Proposed Toeplitz–Hankel structured covariance reconstruction**Require:** 
Received data matrix X, source number *K***Ensure:** 
Estimated DOAs $\hat{\theta }$  1:Compute the sample covariance matrix.  2:Construct the difference coarray and perform redundancy-aware lag averaging.  3:Recover missing lags using PCHIP interpolation and reconstruct the zero-lag component.  4:Form the virtual Toeplitz covariance matrix.  5:Apply iterative PSD projection and Toeplitz diagonal averaging.  6:Construct the Hankel matrix and perform truncated SVD refinement.  7:Recover the refined covariance sequence and construct the final covariance matrix.  8:**for** each FBSS scale **do**  9:      Compute the MUSIC spectrum.10:**end for**11:Fuse the spectra from different smoothing scales.12:Detect spectral peaks and estimate DOAs.

### 3.5. Cramér–Rao Bound Analysis

The Cramér–Rao Bound (CRB) is used as a theoretical reference to evaluate the estimation accuracy of the compared methods. Since the considered noise is sensor-dependent and nonuniform, the CRB is calculated after noise whitening so that the effect of the diagonal noise covariance can be incorporated into the bound.

From the received signal model in (4), the nonuniform noise covariance is

(46) $$R_{n}=\mathrm{diag}\left\{\sigma_{1}^{2},\sigma_{2}^{2},\ldots ,\sigma_{L}^{2}\right\}.$$

Define the whitening matrix as

(47) $$W_{n}=R_{n}^{-1/2}.$$

Then the whitened observation model can be written as

(48) $$\bar{x}\left(t\right)=W_{n}x\left(t\right)=\bar{A}\left(\theta \right)s\left(t\right)+\bar{n}\left(t\right),$$

where

(49) $$\bar{A}\left(\theta \right)=W_{n}A\left(\theta \right),\;E\left\{\bar{n}\left(t\right){\bar{n}}^{H}\left(t\right)\right\}=I.$$

For the *k*th source, the derivative of the steering vector with respect to the DOA is

(50) $$\frac{\partial a(\theta_{k})}{\partial \theta_{k}}=-j\pi \cos\theta_{k}{\left[p_{1}e^{-j\pi p_{1}\sin\theta_{k}},\ldots ,p_{L}e^{-j\pi p_{L}\sin\theta_{k}}\right]}^{T}.$$

The corresponding whitened derivative matrix is defined as

(51) $$\bar{D}=W_{n}\left[\frac{\partial a(\theta_{1})}{\partial \theta_{1}},\frac{\partial a(\theta_{2})}{\partial \theta_{2}},\ldots ,\frac{\partial a(\theta_{K})}{\partial \theta_{K}}\right].$$

Let the orthogonal projection matrix onto the complement of the whitened array manifold be

(52) $$P_{\bar{A}}^{⊥}=I-\bar{A}{\left({\bar{A}}^{H}\bar{A}\right)}^{-1}{\bar{A}}^{H}.$$

For deterministic source signals, the Fisher information matrix for the DOA vector can be expressed as

(53) $$F=2T\;ℜ\left\{\left({\bar{D}}^{H}P_{\bar{A}}^{⊥}\bar{D}\right)⊙R_{s}^{T}\right\},$$

where *T* is the number of snapshots, $R_{s}$ is the source covariance matrix, and ⊙ denotes the Hadamard product. The CRB matrix is obtained by inverting the Fisher information matrix:

(54) $$C_{\mathrm{CRB}}=F^{-1}.$$

The RMSE-type CRB plotted in the simulations is then calculated as

(55) $${\mathrm{CRB}}_{\mathrm{RMSE}}=\sqrt{\frac{1}{K}\mathrm{tr}\left(C_{\mathrm{CRB}}\right)}.$$

**Figure 1:**
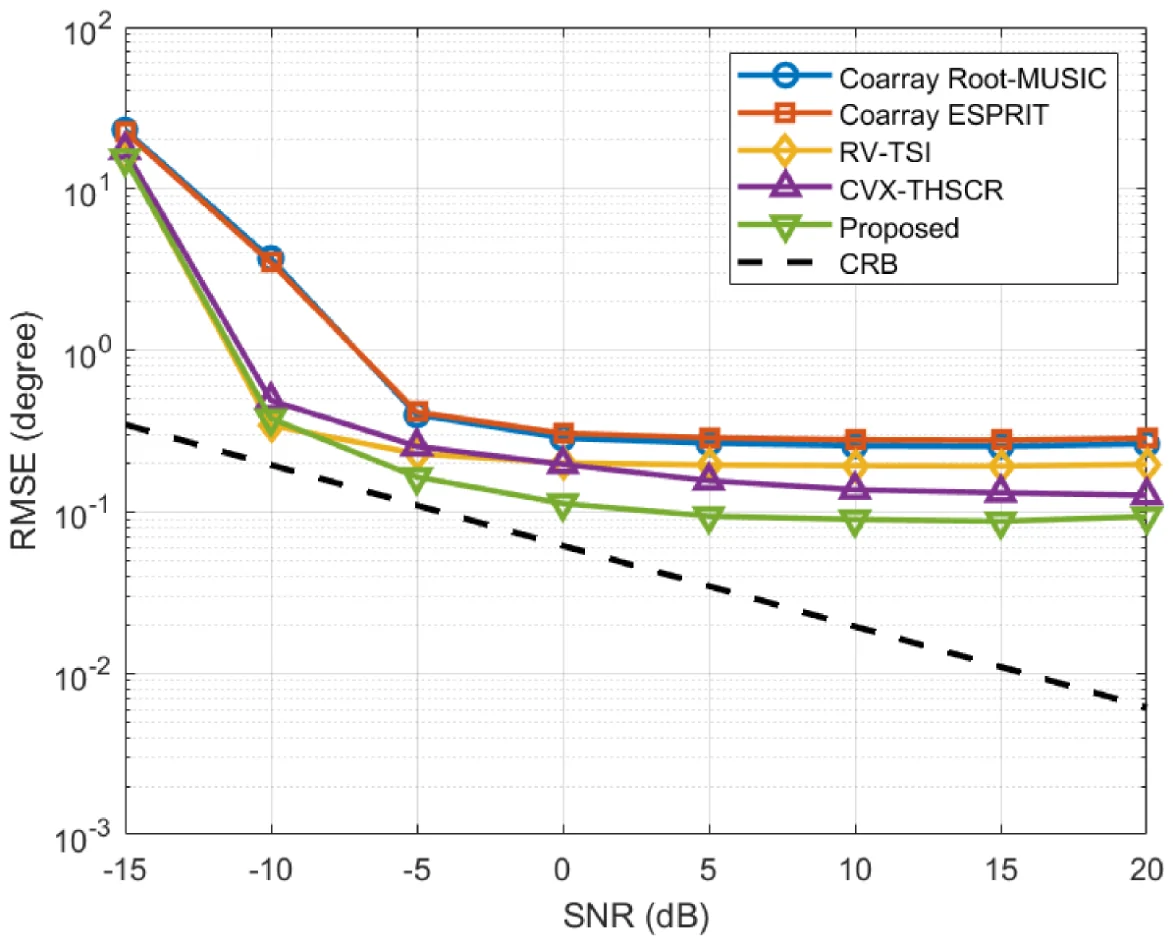
RMSE versus SNR for different DOA estimation methods.

**Figure 2:**
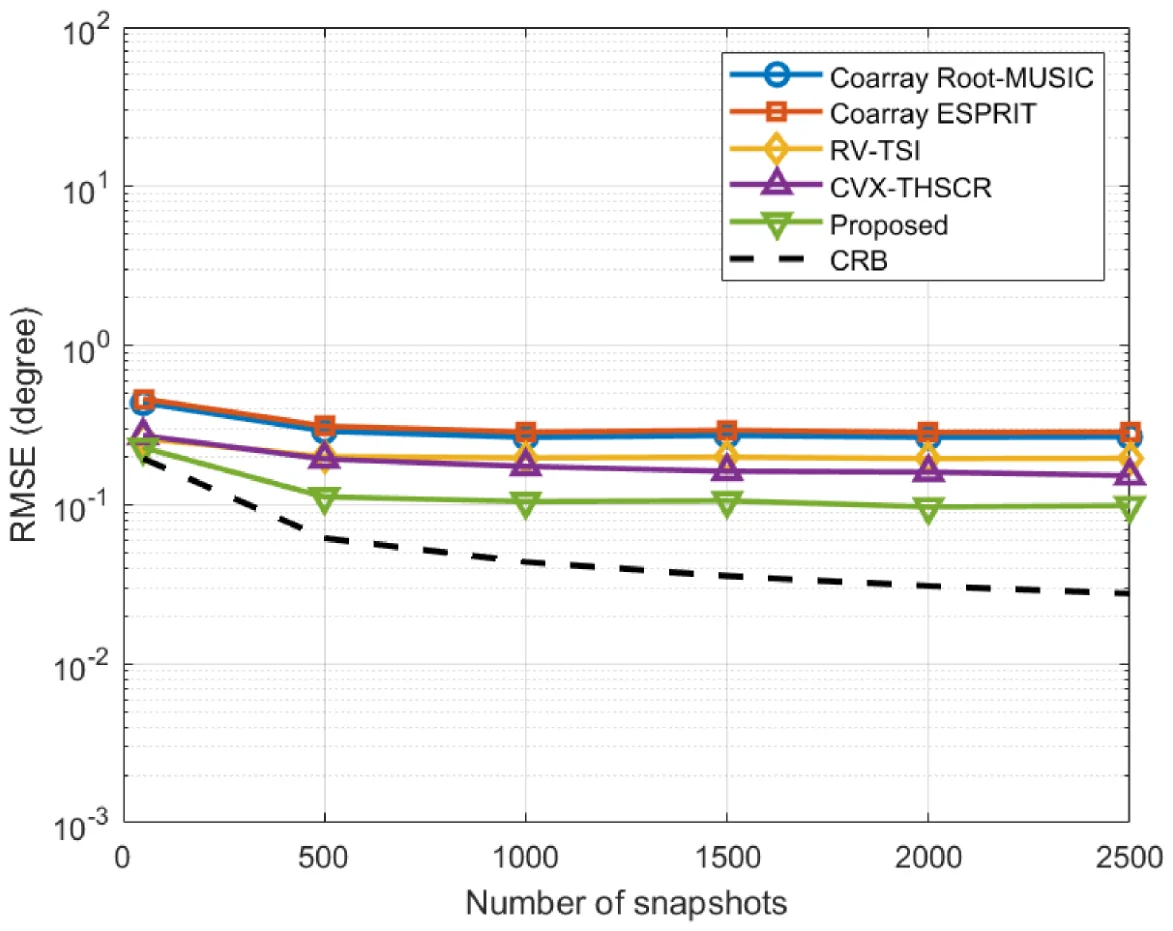
RMSE versus snapshots for different DOA estimation methods.

This bound provides the minimum achievable RMSE of any unbiased DOA estimator under the same array geometry, source configuration, snapshot number, and noise-power setting. Therefore, the CRB curves in Figure 1 and Figure 2 are used only as theoretical benchmarks rather than as practical estimation algorithms.

## 4. Simulation Setup

In the simulations, the number of incident sources *K* is assumed to be known when the compared DOA estimators are executed. This setting is commonly used in controlled performance evaluation because it allows the RMSE, runtime, and complexity of different DOA estimation algorithms to be compared under the same source configuration. In the proposed method, *K* is used in two main steps: the retained rank of the Hankel truncated-SVD refinement is selected according to $r_{h}=K+1$, and the final MUSIC spectrum is searched for *K* dominant peaks.

When the number of sources is unknown in practical applications, the proposed framework can be combined with low-complexity source-number estimation methods before the Hankel refinement and MUSIC peak-search stages. For example, information-theoretic criteria such as AIC and MDL, eigenvalue-gap detection, or SORTE-type eigenvalue methods can be applied to the reconstructed Toeplitz covariance matrix.

All simulations were conducted on a computer equipped with an Intel Core i7-13700KF processor (Intel, Santa Clara, CA, USA), an NVIDIA GeForce RTX 4070 SUPER graphics card (Nvidia, Santa Clara, CA, USA), and 16 GB RAM. The coprime array used throughout the simulations was specified by M=4 and N=5, corresponding to the physical sensor-position set

(56) P={0,4,5,8,10,12,15,16}.

Unless otherwise specified, the nonuniform noise factor was set to α=1.00.

The proposed method was compared with Coarray Root-MUSIC, Coarray ESPRIT, RV-TSI, and CVX-based THSCR. The estimation accuracy was evaluated by the root mean square error (RMSE), and each RMSE point was obtained by averaging the corresponding Monte Carlo trials. Runtime performance was evaluated by recording the total execution time and the average time per Monte Carlo trial.

## 5. Simulation Results and Discussion

### 5.1. RMSE Performance Versus SNR

Figure 1 compares the RMSE curves of Coarray Root-MUSIC, Coarray ESPRIT, RV-TSI, CVX-based THSCR, and the proposed method under different SNRs. The parameter setting is K=2 with DOAs $\left[-10^{\circ },10^{\circ }\right]$, coherence coefficient ρ=0.6, snapshot number T=500, SNR range −15:5:20 dB, and 100 Monte Carlo trials for each SNR point. The CRB is also included as a theoretical lower-bound reference. As the SNR increases, the additive-noise perturbation in the sample covariance matrix is reduced, so the RMSE of all practical algorithms generally decreases and moves toward the CRB trend. The gap between each algorithm and the CRB reflects the combined effects of finite snapshots, coherent-source rank deficiency, missing coarray lags, and unknown nonuniform noise.

Coarray Root-MUSIC and Coarray ESPRIT have relatively simple processing structures. They mainly depend on coarray covariance construction and subspace decomposition, so their curves improve with SNR but remain limited when the virtual covariance is incomplete or biased. RV-TSI uses real-valued transformation and spline interpolation to fill missing virtual lags, and therefore it can improve the continuity of the virtual covariance sequence. However, interpolation alone does not guarantee that the reconstructed covariance matrix is positive semidefinite, nor does it explicitly enhance the low-rank exponential structure associated with the incident sources. Consequently, its RMSE reduction is still limited in the coherent-source and nonuniform-noise environment.

Both CVX-based THSCR and the proposed method use Toeplitz–Hankel structural information, but their reconstruction mechanisms are different. The CVX-based THSCR obtains the Toeplitz covariance through reweighted SDP and recovers the Hankel structure through nuclear-norm optimization. These constraints are mathematically strong, but the final solution depends on the fidelity term, the regularization coefficient, the reweighting process, and the Hankel penalty parameter. In the low-SNR region, the observed lags contain stronger random errors; if these errors are fitted too closely, the reconstructed covariance may inherit noise perturbations. In the high-SNR region, an excessively strong global regularization may smooth the covariance sequence and weaken useful angular information.

The proposed method shows a more favorable RMSE behavior because it applies several targeted corrections before MUSIC estimation. First, the zero-lag component, which is most affected by nonuniform sensor noise, is not used as a normal interpolation sample. Second, bounded zero-lag replacement prevents the diagonal noise power from dominating the Toeplitz covariance. Third, EVD-based PSD projection enforces covariance validity without solving a global SDP. Finally, Hankel truncated SVD keeps the dominant source-related singular components instead of penalizing the whole Hankel matrix by a nuclear norm. These operations preserve the main Toeplitz–Hankel reconstruction idea while reducing the risk of overfitting noisy lags or over-smoothing the angular information. Therefore, the proposed method can be closer to the CRB trend than the other practical methods in Figure 1.

### 5.2. RMSE Performance Versus Snapshots

Figure 2 presents the RMSE curves as the number of snapshots increases. The parameter setting is K=2 with DOAs $\left[-10^{\circ },10^{\circ }\right]$, coherence coefficient ρ=0.6, SNR =0 dB, snapshot range 50–2500, and 100 Monte Carlo trials for each snapshot point. The CRB decreases with the number of snapshots because more observations provide more Fisher information for the DOA parameters. The practical algorithms also benefit from more snapshots, since the sample covariance matrix becomes closer to the theoretical covariance matrix. However, different methods make different use of this improved covariance information.

Coarray Root-MUSIC and Coarray ESPRIT have fast implementation but limited robustness, because the virtual covariance used by them is mainly obtained from direct coarray processing. When the sources are coherent, the effective covariance rank is reduced, and the final subspace separation is sensitive to covariance reconstruction errors. RV-TSI introduces interpolation into the virtual array domain, so the covariance sequence becomes more continuous than that obtained by direct coarray processing. Nevertheless, its interpolated covariance may still contain invalid eigenvalue components and snapshot-induced fluctuations, which explains why its RMSE curve does not fully exploit the increasing snapshot number.

The CVX-based THSCR improves the reconstruction quality by imposing Toeplitz PSD and Hankel low-rank constraints through optimization. However, in the snapshot experiment, its performance is still affected by the balance between data fitting and regularization. When the snapshot number is small, the optimization problem is driven by a noisy sample covariance, and the recovered matrix may be feasible but not optimal for the subsequent spectral peak search. When the snapshot number becomes large, the CVX-based solution still depends on the selected regularization and Hankel penalty; if the penalty is not perfectly matched to the source and noise condition, useful covariance details may be weakened.

The proposed method obtains a smoother and lower RMSE trend because its reconstruction process is more directly matched to the error sources in this experiment. At small snapshot numbers, redundancy-aware lag averaging and PSD projection suppress random covariance fluctuations. At medium and large snapshot numbers, the dominant Hankel singular components become clearer, so truncated SVD can retain the source-related structure while removing residual perturbations. Since the proposed method controls the zero-lag nonuniform-noise bias before reconstruction and avoids global nuclear-norm shrinkage, it can preserve angular information more effectively. This explains why the proposed curve shows a consistent advantage while following the same decreasing tendency indicated by the CRB in Figure 2.

### 5.3. Influence of Source Coherence

**Figure 3:**
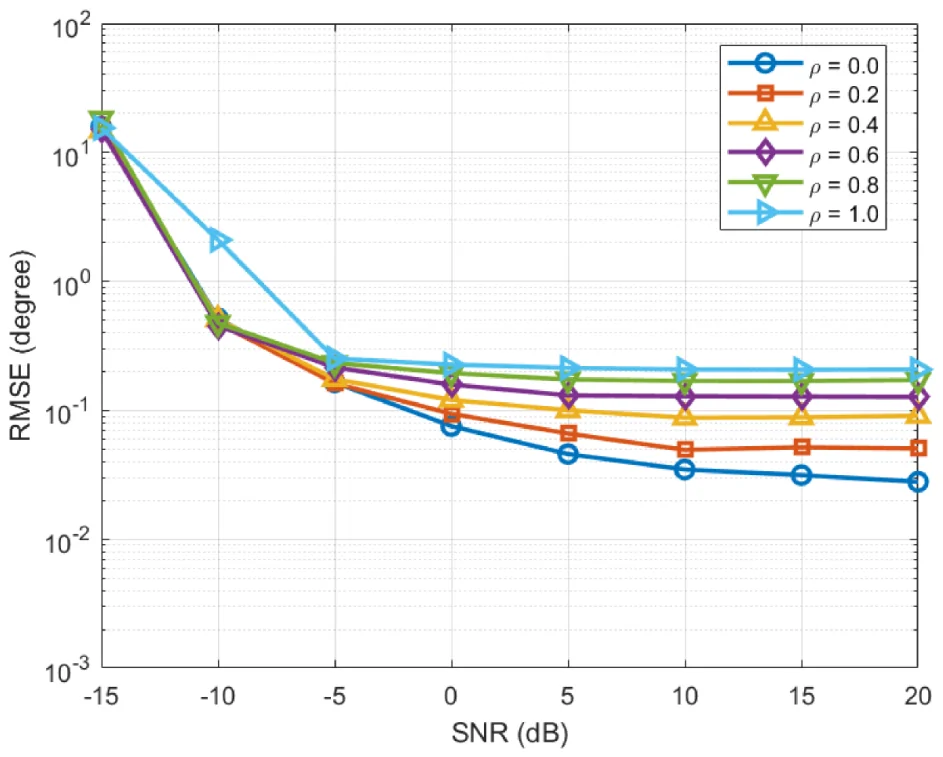
RMSE versus SNR under different coherence coefficients.

**Figure 4:**
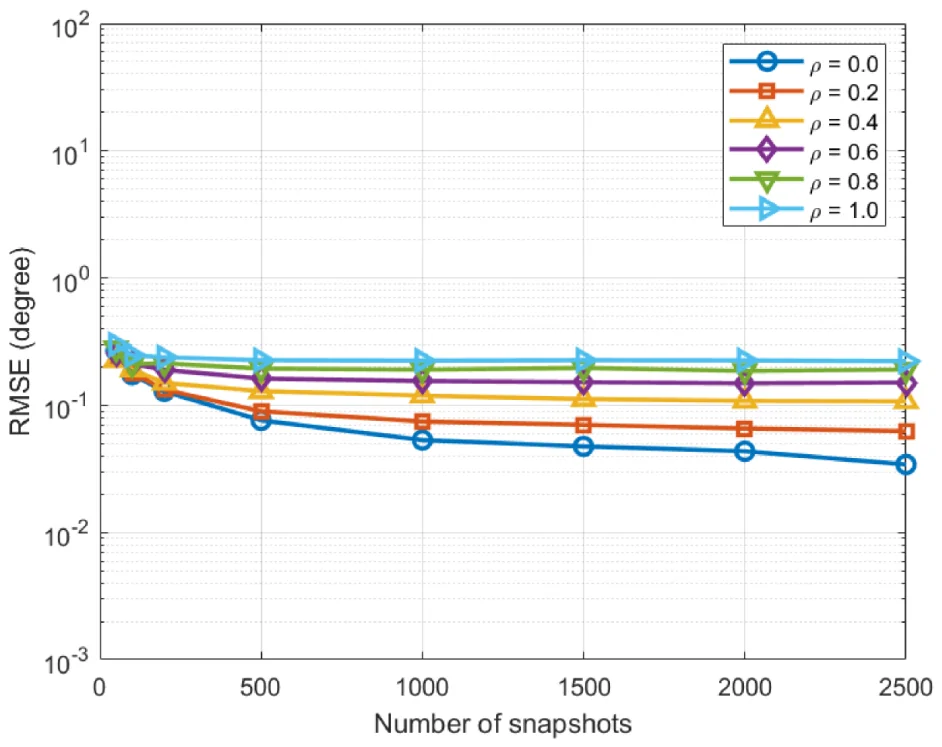
RMSE versus snapshots under different coherence coefficients.

Figure 3 and Figure 4 evaluate the performance of the proposed method under different coherence coefficients. In this experiment, two sources are fixed at $\left[-10^{\circ },10^{\circ }\right]$, and the coherence coefficient is selected from {0,0.2,0.4,0.6,0.8,1.0}. For Figure 3, the number of snapshots is fixed at T=500, the SNR varies from −15 dB to 20 dB with a 5 dB interval, and 100 Monte Carlo trials are used for each point. For Figure 4, the SNR is fixed at 0 dB, the number of snapshots varies from 50 to 2500, and 100 Monte Carlo trials are used.

A larger coherence coefficient means that the source covariance matrix becomes more rank deficient, making the signal subspace more difficult to recover. The curves show that the proposed method can still reduce RMSE as SNR or snapshots increase, which indicates that the reconstruction framework is effective under coherent-source conditions. The reason is that the Hankel truncated-SVD operation strengthens the exponential structure of the virtual covariance sequence, while FBSS restores the rank needed for MUSIC-based estimation. Thus, even when the source signals are highly correlated, the reconstructed covariance can still provide distinguishable spectral information.

The results also reveal that the RMSE gap between different coherence levels becomes more obvious in difficult conditions. Under low SNR or limited snapshots, stronger coherence causes more severe subspace ambiguity. The proposed method alleviates this effect by combining covariance-structure correction and spatial smoothing. Therefore, the figures demonstrate that the proposed method is not only effective for a single coherence setting, but also has good robustness when the source correlation degree changes.

### 5.4. Influence of Nonuniform Noise Level

After analyzing the influence of source coherence, this subsection further investigates the sensitivity of the proposed method to the degree of noise nonuniformity. In practical sparse-array systems, different sensors may have different noise powers because of hardware mismatch, calibration errors, mutual coupling, temperature drift, or environmental interference. According to (12) and (13), the parameter α controls the sensor-dependent noise-power variation. When α=0, all sensors have equal noise power, corresponding to the uniform-noise case. As α increases, the noise-power mismatch among sensors becomes stronger, and the zero-lag component in the difference-coarray domain is more severely affected by the diagonal noise powers. Therefore, this experiment is used to verify whether the proposed zero-lag suppression and structured covariance reconstruction can maintain stable estimation performance under different nonuniform-noise levels.

In this experiment, the nonuniform-noise factor is selected as α={0,0.5,1.0,2.0,4.0}. The tested range 0≤α≤4 covers the transition from the uniform-noise case to a relatively strong nonuniform-noise condition. According to the adopted noise model, α represents the normalized amplitude of sensor-dependent noise-power variation. If the maximum normalized perturbation is considered, the largest sensor noise power is approximately proportional to 1+α. Therefore, the tested range corresponds to a maximum noise-power ratio from 1 to 5, namely an equivalent noise-power variation of approximately 0–6.99 dB, calculated as

(57) $$\Delta_{n}=10\log_{10}\left(1+\alpha \right).$$

Specifically, α=0 denotes the ideal uniform-noise case, while α=0.5, 1.0, 2.0, and 4.0 correspond to progressively stronger nonuniform-noise conditions. These values provide a reasonable robustness-test range because they cover mild, moderate, and severe sensor-dependent noise-power mismatches. Therefore, the selected range is sufficient for evaluating the stability of the proposed method under different degrees of nonuniform noise.

**Figure 5:**
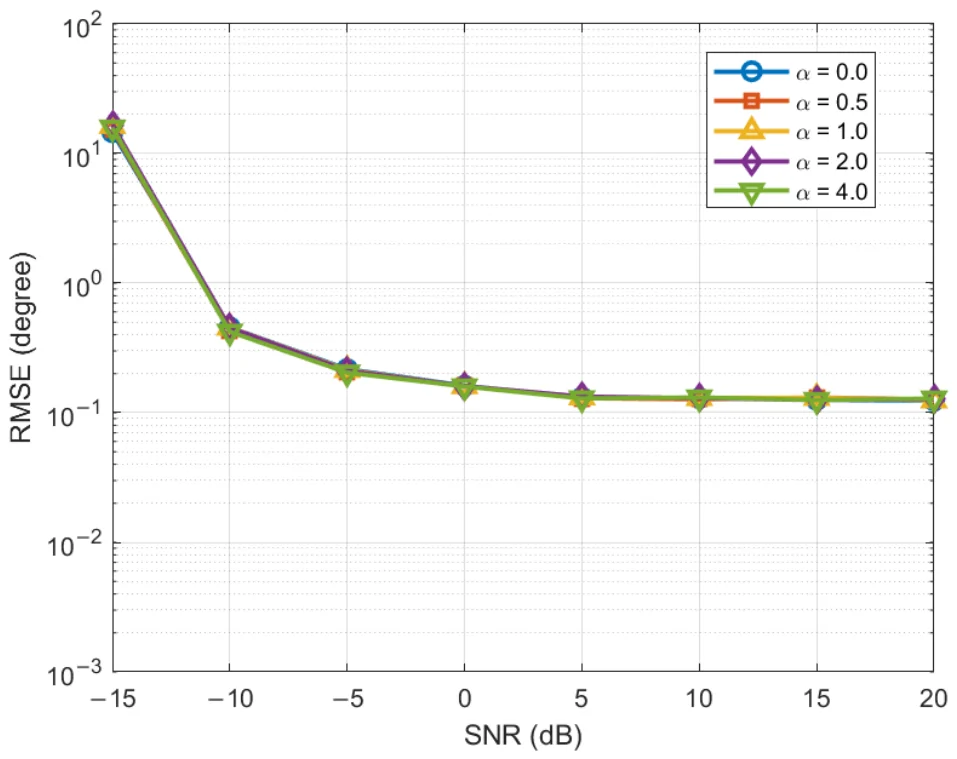
RMSE versus SNR under different nonuniform noise factors.

**Figure 6:**
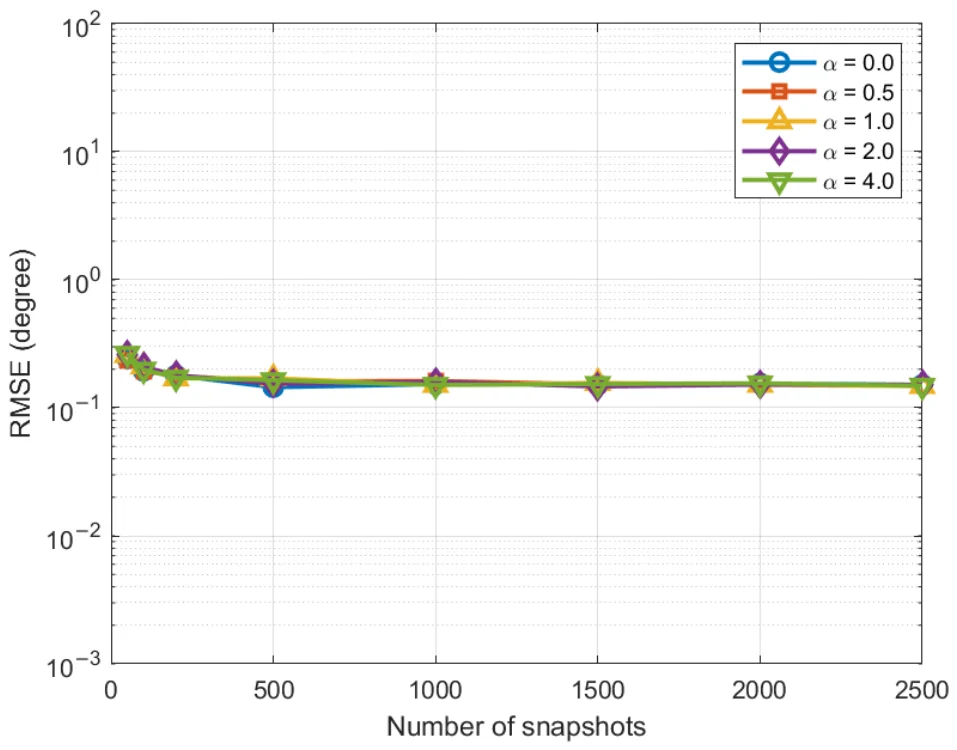
RMSE versus snapshots under different nonuniform noise factors.

Figure 5 and Figure 6 show the RMSE performance of the proposed method under different values of α. For Figure 5, the number of snapshots is fixed at T=500, the coherence coefficient is ρ=0.6, and the SNR varies from −15 dB to 20 dB. For Figure 6, the SNR is fixed at 0 dB, the coherence coefficient is ρ=0.6, and the number of snapshots varies from 50 to 2500.

As shown in Figure 5, the RMSE curves corresponding to different α values are very close to each other over the tested SNR range. At low SNR, the RMSE is mainly dominated by additive noise and finite-snapshot covariance estimation errors, so the influence of the noise nonuniformity factor is relatively small compared with the overall noise level. As the SNR increases, all curves decrease rapidly and then tend to a stable region. The close agreement among the curves indicates that the proposed method is not sensitive to the selected value of α in the SNR experiment.

Figure 6 further confirms this observation from the snapshot perspective. When the number of snapshots increases, the sample covariance estimation becomes more stable. The RMSE curves for α=0, 0.5, 1.0, 2.0, and 4.0 remain close to each other, showing that the proposed method can maintain stable estimation accuracy under different degrees of sensor-dependent noise power. Although a larger α introduces stronger zero-lag contamination, the zero-lag suppressed interpolation and bounded zero-lag replacement prevent the diagonal noise power from dominating the reconstructed Toeplitz covariance.

These results demonstrate that the proposed method has good robustness to the nonuniform-noise factor. In the tested range 0≤α≤4, which corresponds to approximately 0–6.99 dB equivalent noise-power variation, the proposed method maintains stable RMSE performance in both the SNR and snapshot experiments. This confirms the effectiveness of the proposed zero-lag suppression and structured covariance reconstruction strategy for DOA estimation under unknown nonuniform noise.

### 5.5. Influence of the Number of Sources

**Figure 7:**
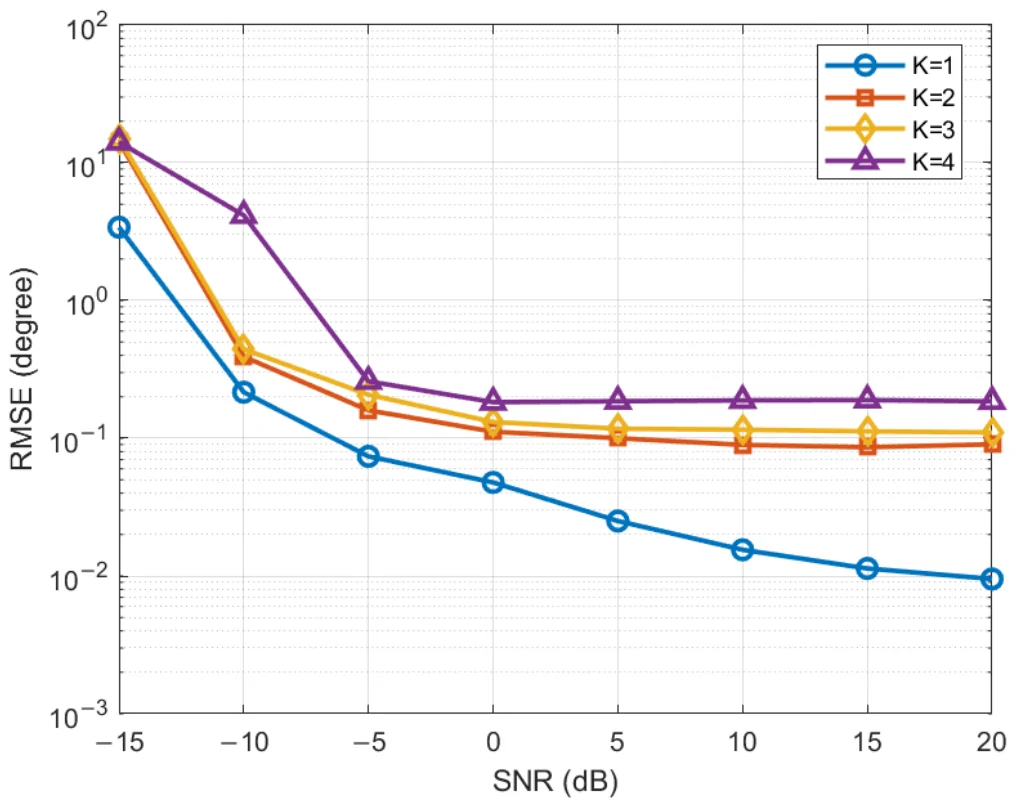
RMSE versus SNR for different numbers of sources.

**Figure 8:**
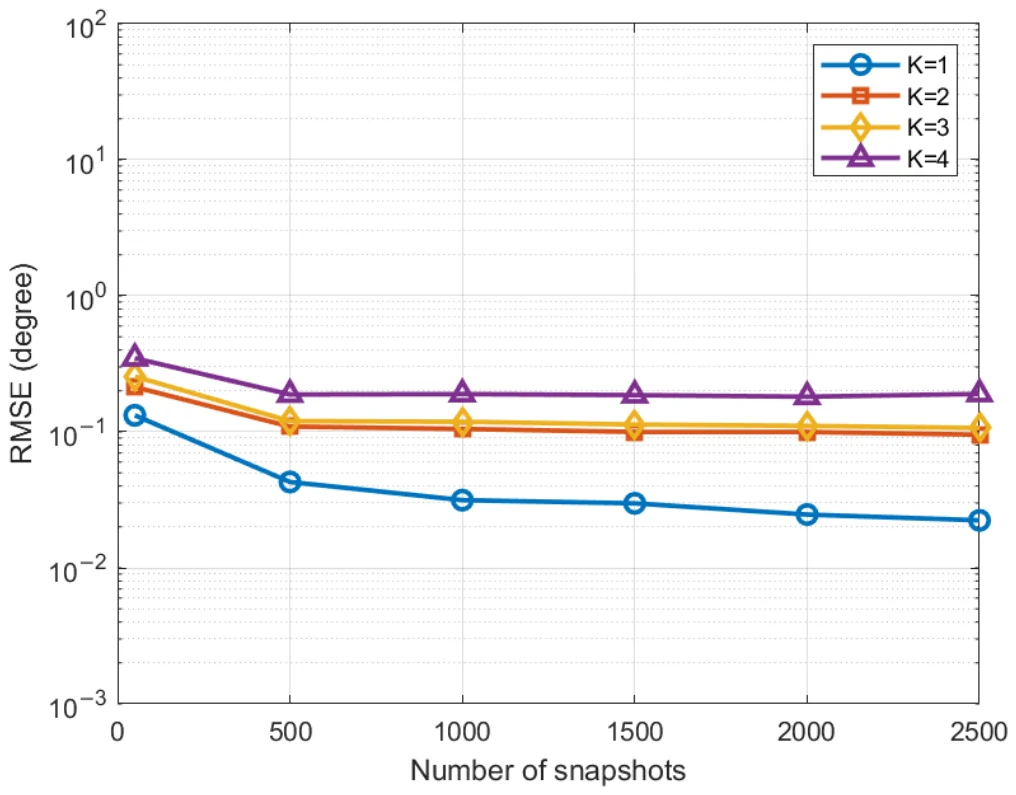
RMSE versus snapshots for different numbers of sources.

Figure 7 and Figure 8 show the RMSE performance for different numbers of sources. In the SNR experiment, the reference two-source setting is K=2 with DOAs $\left[-10^{\circ },10^{\circ }\right]$, the coherence coefficient is ρ=0.6, the number of snapshots is T=500, the SNR range is −15:5:20 dB, and 100 Monte Carlo trials are used. In the snapshot experiment, the SNR is fixed at 0 dB, the snapshot number varies from 50 to 2500, and 100 Monte Carlo trials are used. These parameter settings ensure that the performance variation mainly reflects the effect of increasing the number of targets.

As the source number increases, the dimension of the signal subspace increases and the angular spectrum becomes more crowded. This makes peak separation more difficult and usually increases RMSE. Even under this more difficult condition, the proposed method retains a clear estimation capability because the coprime array provides an enlarged virtual aperture and the Toeplitz–Hankel reconstruction improves the continuity and validity of the virtual covariance.

The performance trend also supports the design of the Hankel refinement stage. Since the Hankel matrix formed from the covariance sequence contains source-related exponential components, the retained rank should depend on the number of sources. In the proposed method, the retained Hankel rank is selected as K+1, which allows the dominant source components to be preserved while suppressing covariance perturbation. Therefore, when *K* increases, the method can adapt the refinement strength instead of using a fixed low-rank model. This explains why the proposed method maintains robustness in the multi-source experiments.

### 5.6. MUSIC Spectrum Analysis

**Figure 9:**
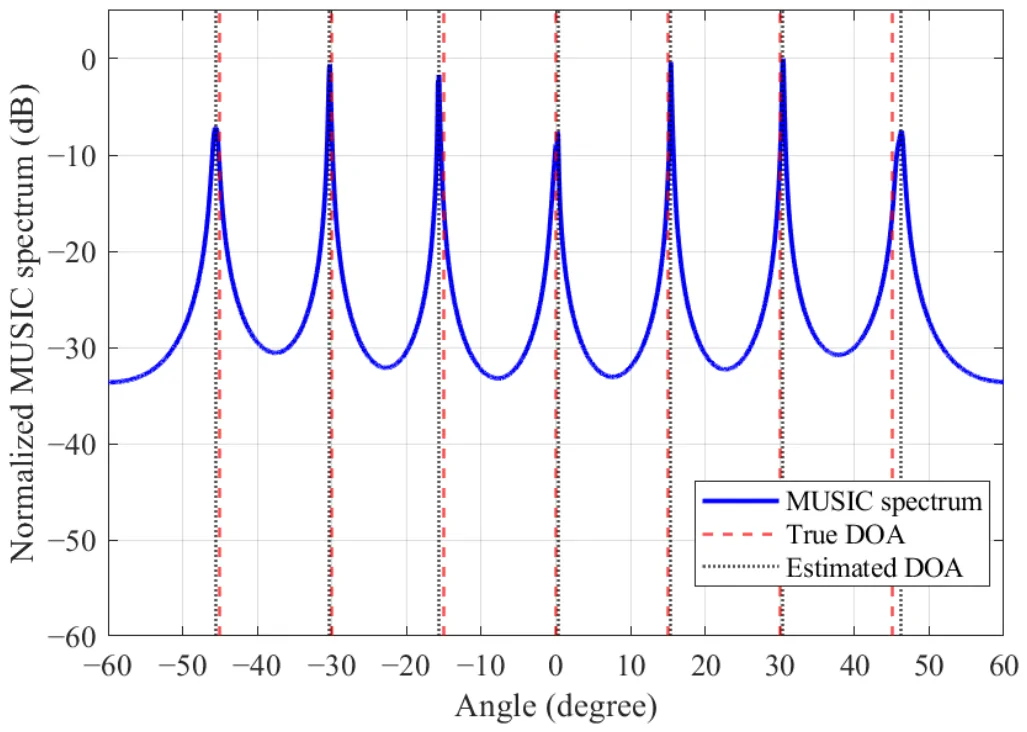
MUSIC spectrum of the proposed method for K=7 sources.

Figure 9 gives the MUSIC spectrum obtained after the proposed covariance reconstruction. In this experiment, seven coherent sources are located at

(58) $$\theta =[-45^{\circ },-30^{\circ },-15^{\circ },0^{\circ },15^{\circ },30^{\circ },45^{\circ }],$$

where the coherence coefficient is ρ=0.6, the number of snapshots is T=1000, and the SNR is 10 dB. The minimum angular spacing between adjacent sources is $15^{\circ }$, which provides a challenging multi-target scenario for evaluating the angular resolution capability of the proposed method.

The main information in Figure 9 is the number, location, and sharpness of the spectral peaks. The spectrum forms seven distinguishable peaks around the true DOA positions, and no obvious false peak dominates the spectrum. The peak positions correspond well to the preset source angles, showing that the proposed covariance reconstruction provides a clean and structured virtual covariance matrix even when multiple coherent targets coexist. The suppressed sidelobe level also indicates that the noise subspace is well separated from the source subspace after Toeplitz PSD projection, Hankel truncated-SVD refinement, and multi-scale FBSS processing.

The spectral behavior directly reflects the advantage of the proposed method. Zero-lag suppression prevents the unknown nonuniform noise power from weakening the nonzero-lag angular information. Toeplitz PSD projection ensures that the matrix used for eigendecomposition is a valid covariance matrix. Hankel truncated-SVD refinement concentrates the dominant exponential components corresponding to the seven source directions. Multi-scale FBSS further stabilizes the spectrum under source coherence. Therefore, Figure 9 demonstrates that the proposed method has strong multi-target resolution capability and can obtain clear MUSIC peaks under the challenging setting of K=7 coherent sources.

To further respond to the case where the number of sources is larger than the number of physical sensors, an additional MUSIC-spectrum experiment is conducted. For the adopted coprime array with M=4 and N=5, the number of physical sensors is L=8. In this experiment, the number of incident sources is increased to K=9, which is larger than the number of physical sensors. The true DOAs are set as

(59) $$\theta =[-60^{\circ },-45^{\circ },-30^{\circ },-15^{\circ },0^{\circ },15^{\circ },30^{\circ },45^{\circ },60^{\circ }].$$

The coherence coefficient is set to ρ=0.6, the number of snapshots is set to T=512, and the SNR is 10 dB. The nonuniform-noise coefficient is set to α=0.25. This value is selected according to the receiver noise-figure variation reported in the TI AWR1243 data sheet [29], where the receiver noise figure varies with the programmed receiver gain. By converting a moderate receiver noise-figure variation into the linear noise-power domain, a 1 dB-level variation corresponds to approximately $10^{1/10}-1\approx 0.26$, and therefore α=0.25 is adopted as a practical hardware-data-oriented nonuniform-noise setting. In addition, the snapshot number T=512 is consistent with a typical TI mmWave raw-data acquisition scale, where AWR1243 transfers raw ADC samples through the CSI-2 interface and the AWR1243BOOST platform can be used together with DCA1000/mmWave Studio for raw radar data capture [30].

**Figure 10:**
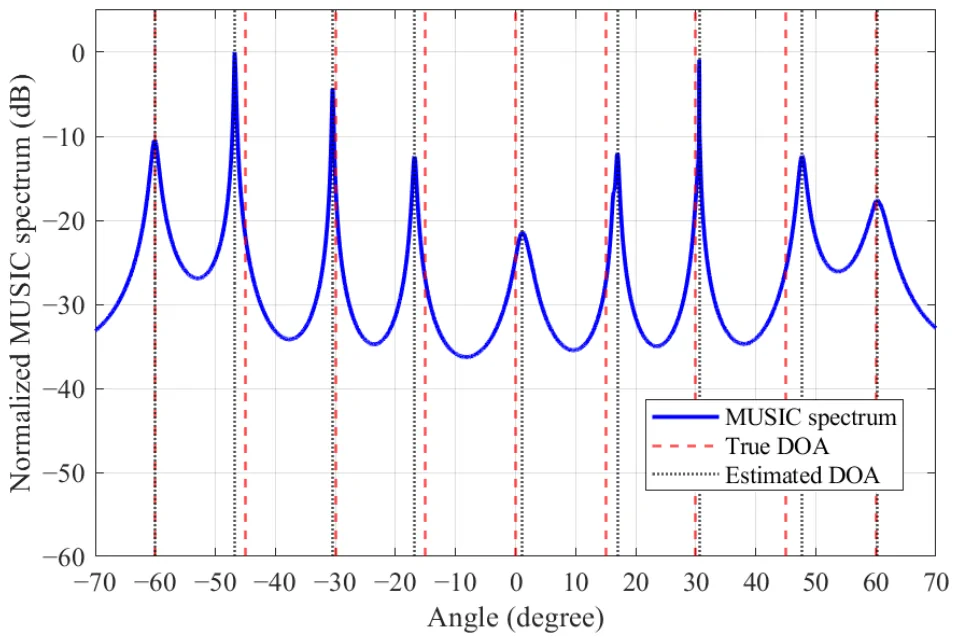
MUSIC spectrum of the proposed method for K=9 sources.

As shown in Figure 10, the proposed method produces nine distinguishable spectral peaks around the true DOA positions. Although only eight physical sensors are used, the coprime difference coarray provides an enlarged virtual aperture and more virtual degrees of freedom. By combining redundancy-aware lag averaging, zero-lag suppressed interpolation, Toeplitz PSD projection, Hankel truncated-SVD refinement, and multi-scale FBSS-MUSIC, the proposed method can reconstruct a structured virtual covariance matrix that contains sufficient angular information for resolving the nine sources under the TI-data-based setting of α=0.25 and T=512.

### 5.7. Scatter Distribution of Estimated DOAs

**Figure 11:**
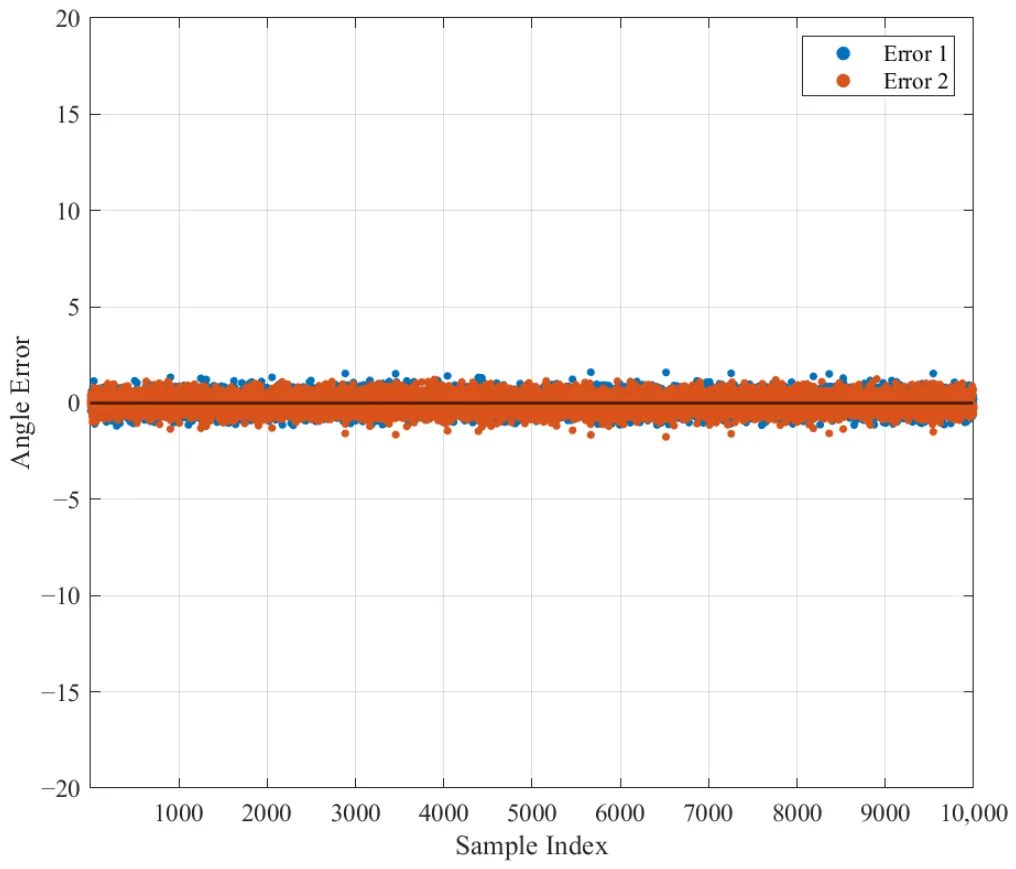
Estimated DOA scatter distribution of the proposed method.

Figure 11 shows the scatter distribution of the estimated DOAs obtained by the proposed method. In this experiment, two source directions are randomly generated within $\left[-50^{\circ },50^{\circ }\right]$, the coherence coefficient is ρ=0.5, the number of snapshots is T=1000, the SNR is 5 dB, and a total of 10,000 random samples are used. Different from the fixed-angle RMSE experiments, this scatter experiment evaluates the behavior of the proposed method over a wide angular region and under many random source configurations.

The scatter points are mainly distributed near the ideal diagonal relationship between the true and estimated angles, indicating that the proposed method maintains stable estimation accuracy for randomly located sources in the whole tested angular interval. The absence of obvious large-scale outliers shows that the proposed covariance reconstruction does not frequently produce wrong spectral peaks, even when the source angles change over a broad range. This result demonstrates the statistical stability and angular generalization capability of the proposed method.

The advantage of the proposed method is reflected in the compact distribution of the estimated points. The zero-lag suppressed interpolation reduces the influence of nonuniform noise, the Toeplitz PSD projection improves covariance validity, the Hankel truncated-SVD refinement suppresses residual perturbations, and multi-scale FBSS-MUSIC stabilizes the final peak search. As a result, most estimates remain close to the true angles. Therefore, Figure 11 provides sample-level evidence that the proposed method has good robustness and repeatability, rather than only good average RMSE performance.

### 5.8. Runtime Analysis

Runtime performance was evaluated under K=2 with DOAs $\left[-10^{\circ },10^{\circ }\right]$, coherence coefficient ρ=0.6, snapshot number T=500, SNR =0 dB, and nonuniform noise factor α=1.00. The number of Monte Carlo trials was selected as 10, 30, 50, and 80.

**Figure 12:**
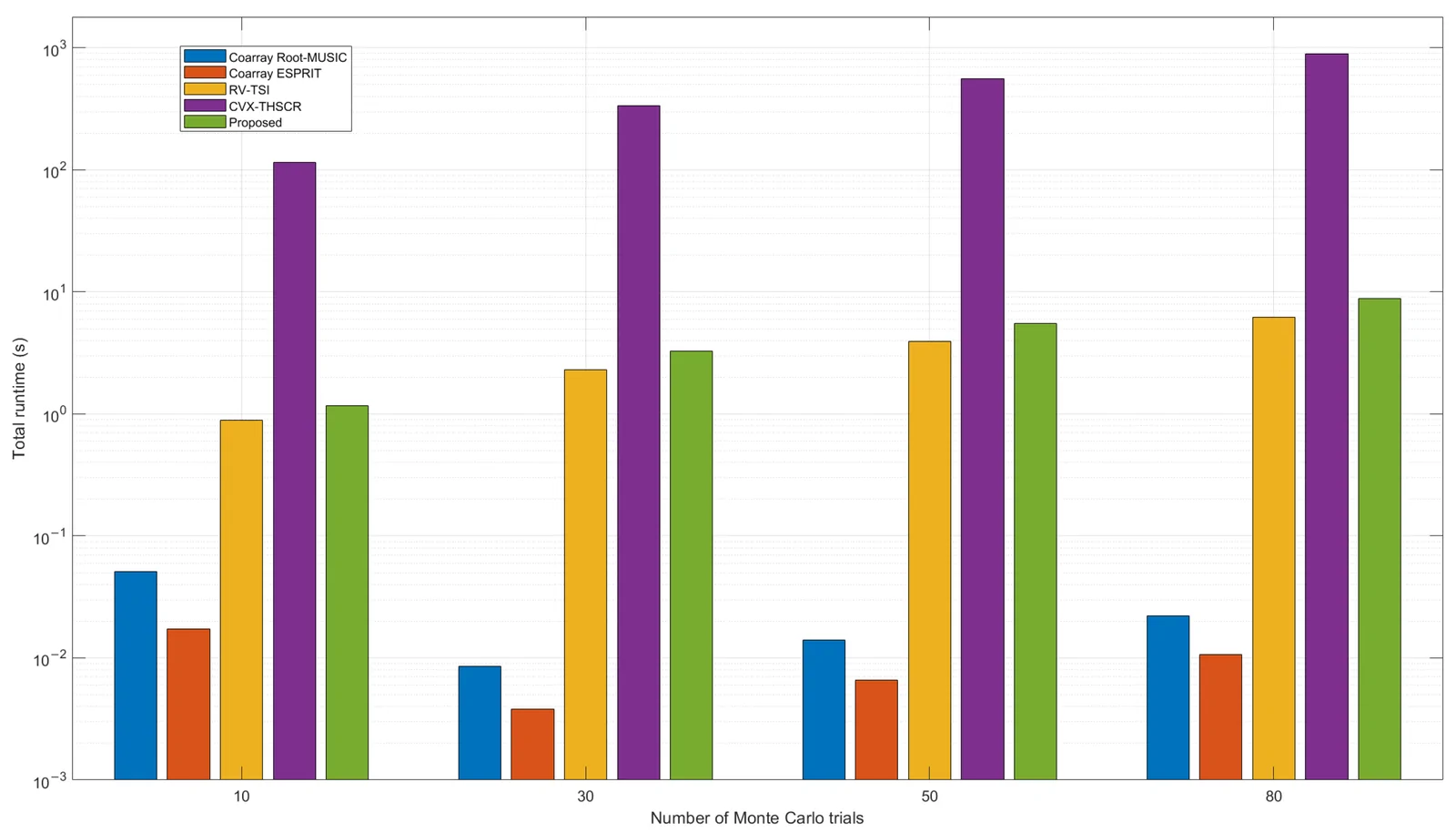
Runtime comparison under different Monte Carlo trials.

Figure 12 illustrates the total runtime of each method, while Table 1 reports the average runtime per Monte Carlo trial. These two results describe different aspects of computational efficiency. The total-runtime curves show how the computational burden accumulates as the number of repeated trials increases, whereas the average-runtime table reflects the intrinsic computational cost of one trial for each method.

As shown in Figure 12, the total runtime of all methods increases approximately linearly with the number of Monte Carlo trials. This is reasonable because each Monte Carlo trial repeats signal generation, sample covariance estimation, virtual covariance reconstruction, and DOA estimation. Among the compared methods, CVX-based THSCR has the steepest growth trend and reaches approximately 897.57 s when MC =80, indicating that the optimization-based reconstruction dominates the total execution time.

The reason for this high runtime is that CVX-based THSCR contains two time-consuming optimization stages. In the Toeplitz reconstruction stage, a semidefinite programming problem is solved to enforce the Toeplitz positive semidefinite covariance constraint. In the Hankel enhancement stage, nuclear-norm optimization is used to promote low-rank structure. Both stages rely on iterative convex solvers, repeated matrix factorizations, and convergence checking, which significantly increase the computational cost.

In contrast, the proposed method replaces these optimization-based operations with direct matrix-processing steps. PCHIP interpolation and bounded zero-lag replacement are used for missing-lag recovery; EVD-based PSD projection and Toeplitz diagonal averaging are used for covariance-validity enforcement; truncated singular value decomposition is used for Hankel spectral refinement; and multi-scale FBSS-MUSIC is used for final DOA estimation. These operations preserve the main Toeplitz–Hankel structured reconstruction mechanism but avoid repeated interior-point optimization.

According to Table 1, the proposed method requires approximately 0.11 s per trial, whereas CVX-based THSCR requires approximately 11.2 s per trial. Therefore, the proposed method achieves an acceleration factor of about 101.78 under the tested setting. Compared with RV-TSI, the proposed method has a slightly higher average runtime because it includes PSD projection, Hankel refinement, and multi-scale FBSS-MUSIC after interpolation. However, this additional computational cost is used to improve covariance validity, suppress residual perturbations, and enhance coherent-source resolution. Thus, the proposed method provides a more balanced trade-off between estimation accuracy and computational efficiency.

## 6. Computational Complexity Analysis

This section gives the complexity derivation and formula-based comparison of different methods. Let *L* denote the number of physical sensors, *T* the number of snapshots, *Q* the virtual ULA covariance length, *K* the number of sources, *G* the number of angular grid points, $J_{p}$ the number of PSD projection iterations, *S* the number of FBSS scales, $p_{s}$ the subarray length at the *s*th scale, and $L_{h}\times R_{h}$ the size of the Hankel matrix. For the proposed method, the sample covariance matrix requires

(60) $$C_{\mathrm{cov}}=O\left(L^{2}T\right),$$

because it multiplies an L×T data matrix by a T×L matrix. The coarray lag-averaging step visits all sensor pairs, so its complexity is

(61) $$C_{\mathrm{lag}}=O\left(L^{2}\right).$$

The zero-lag suppressed interpolation operates on the virtual covariance sequence and has approximately linear complexity,

(62) $$C_{\mathrm{int}}=O\left(Q\right),$$

while Toeplitz matrix construction requires

(63) $$C_{\mathrm{toep}}=O\left(Q^{2}\right).$$

The main cost of the Toeplitz PSD projection is the eigendecomposition of a Q×Q matrix. Since Toeplitz diagonal averaging is only $O(Q^{2})$, the cost of $J_{p}$ projection iterations is approximated by

(64) $$C_{\mathrm{psd}}=J_{p}\left[O\left(Q^{3}\right)+O\left(Q^{2}\right)\right]\approx O\left(J_{p}Q^{3}\right).$$

For Hankel refinement, if truncated SVD retains $r_{h}=K+1$ dominant singular components, the complexity is

(65) $$C_{\mathrm{hsvd}}=O\left(L_{h}R_{h}r_{h}\right)=O\left(L_{h}R_{h}\left(K+1\right)\right).$$

If MATLAB R2024a economy SVD is used directly, a conservative upper bound is

(66) $$C_{\mathrm{hsvd}}=O\left(L_{h}R_{h}\min\left(L_{h},R_{h}\right)\right).$$

Anti-diagonal averaging is lower order with complexity $O(L_{h}R_{h})$. For the multi-scale FBSS-MUSIC stage, the *s*th scale requires smoothing, eigendecomposition, and grid search. Its cost can be written as

(67) $$C_{\mathrm{fbss}}=\sum_{s=1}^{S}\left[O\left(\left(Q-p_{s}+1\right)p_{s}^{2}\right)+O\left(p_{s}^{3}\right)+O\left(Gp_{s}^{2}\right)\right].$$

Since $p_{s}$ is proportional to *Q* in the implementation, this term can be simplified as

(68) $$C_{\mathrm{fbss}}\approx O\left(SQ^{3}+SGQ^{2}\right).$$

Therefore, the total complexity of the proposed method is

(69) $$C_{\mathrm{prop}}\approx O\left(L^{2}T+J_{p}Q^{3}+C_{\mathrm{hsvd}}+SQ^{3}+SGQ^{2}\right).$$

This formula shows that the proposed method is mainly dominated by EVD-based PSD projection, Hankel SVD, and multi-scale FBSS-MUSIC. All these operations are explicit matrix operations. Therefore, the proposed method avoids the high-order and solver-dependent complexity of CVX-based SDP and nuclear-norm optimization.

From Table 2, the proposed method is more complex than Coarray Root-MUSIC and Coarray ESPRIT because it performs additional covariance reconstruction. However, these additional operations are the reason for its stronger estimation performance under coherent sources and unknown nonuniform noise. Compared with RV-TSI, the proposed method adds PSD projection and Hankel refinement, which increases the computational cost but improves the validity and low-rank spectral structure of the reconstructed covariance. Compared with CVX-based THSCR, the proposed method has a major computational advantage because it replaces SDP and nuclear-norm optimization with EVD, truncated SVD, and direct MUSIC search. This complexity difference is consistent with the runtime experiment, where the proposed method reduces the average runtime from about 11.2 s per trial to about 0.11 s per trial while preserving the Toeplitz–Hankel structured reconstruction advantage.

## 7. Conclusions

This paper proposed a Toeplitz–Hankel structured covariance reconstruction method for DOA estimation of coherent sources using coprime arrays under unknown nonuniform noise. The method combines redundancy-aware difference-coarray lag averaging, zero-lag suppressed interpolation, Toeplitz PSD projection, Hankel truncated-SVD refinement, and multi-scale FBSS-MUSIC. The zero-lag suppression strategy reduces the bias caused by unknown nonuniform sensor noise, while the Toeplitz and Hankel operations improve the physical validity and spectral structure of the reconstructed virtual covariance matrix. Simulation results show that the proposed method achieves better estimation accuracy and stability than Coarray Root-MUSIC, Coarray ESPRIT, and RV-TSI. Compared with CVX-based THSCR, the proposed method avoids SDP and nuclear-norm optimization and reduces the average runtime from about 11.2 s per trial to about 0.11 s per trial in the tested runtime experiment.

## Figures and Tables

**Table 1:** Average runtime per Monte Carlo trial (s).

Method	MC = 10	MC = 30	MC = 50	MC = 80
Coarray Root-MUSIC	0.005089	0.000284	0.000279	0.000275
Coarray ESPRIT	0.001721	0.000127	0.000131	0.000133
RV-TSI	0.089274	0.077054	0.078497	0.078019
CVX-based THSCR	11.494242	11.188058	11.164516	11.219601
Proposed method	0.116461	0.109680	0.110532	0.110229

**Table 2:** Computational complexity formulas of different methods.

Method	Computational Complexity Formula
Coarray Root-MUSIC	$O(L^{2}T+L^{2}+Q^{3})$
Coarray ESPRIT	$O(L^{2}T+L^{2}+Q^{3})$
RV-TSI	$O(L^{2}T+L^{2}+Q^{2}+GQ^{2})$
CVX-based THSCR	$O(L^{2}T+I_{\mathrm{cvx}}C_{\mathrm{SDP}}\left(Q\right)+C_{\mathrm{NN}}\left(L_{h},R_{h}\right))$
Proposed method	$O(L^{2}T+J_{p}Q^{3}+C_{\mathrm{hsvd}}+SQ^{3}+SGQ^{2})$

## Data Availability

The simulation code and data used to support the findings of this study are available from the corresponding author upon reasonable request.
